# A comparative analysis of socioeconomic inequities in stunting: a case of three middle-income African countries

**DOI:** 10.1186/s13690-018-0320-2

**Published:** 2018-12-10

**Authors:** Coretta M. P. Jonah, Winnie C. Sambu, Julian D. May

**Affiliations:** 1 DST-NRF Centre of Excellence in Food Security, Institute for Social Development, University of the Western Cape, School of Government Building, Robert Sobukwe Road/Private Bag X17, Bellville, 7535 South Africa; 2 Children’s Institute, University of Cape Town, 46 Sawkins Road, Rondebosch, 7700 South Africa

**Keywords:** Inequality, Stunting, Children, Malnutrition, Sub-Saharan Africa, Concentration indices, Concentration curves, Middle-income, Ghana, Kenya, Zambia

## Abstract

**Background:**

Despite increased economic growth and development, and existence of various policies and interventions aimed at improving food security and nutrition, majority of countries in sub-Saharan Africa have very high levels of child malnutrition. The prevalence of stunting, an indicator of chronic malnutrition, is especially high.

**Methods:**

In this paper, we use Demographic and Health Survey datasets from three countries in the region that obtained middle-income status over the last decade (Ghana, Kenya and Zambia), to provide a comparative quantitative assessment of stunting levels, and examine patterns in stunting inequalities between 2007 and 2014.

**Results:**

Our analyses reveal that stunting rates decreased in all three countries over the study period, but are still high. In Zambia, 40% of under 5-year olds are stunted, compared to 26% in Kenya and 19% in Ghana. In all three countries, male children and those living in the poorest households have significantly higher levels of stunting. We also observe stark inequalities across socio-economic status, and show that these inequalities have increased over time.

**Conclusions:**

Our results reveal that even with economic gains at the national level, there is need for continued focus on improving the socio-economic levels of the poorest households, if child nutritional outcomes are to improve.

## Background

Globally, countries may be classified into three-income groups - low, middle or high - based on per capita Gross National Income (GNI). Though this classification has been criticised as being narrow [1], the achievement of middle-income status is often seen as an outcome of sustained economic growth fuelled by increased investment in all sectors, including human capital, and improvements in productivity. Thus, an upward movement based on this classification is viewed both nationally and internationally as indicative of economic progress. Such progress is expected to have positive impacts on the welfare of a country’s population, for example through increases in employment opportunities that lead to higher disposable incomes for households. Improvements in other dimensions of well-being such as health and education are also expected [2, 3]. Additionally, households are expected to benefit from these improvements through better nutritional outcomes for both children and adults.

However, while middle income status could be an indicator of improved welfare, the reality is quite different. Over 70% of the world’s poor can be found in middle income economies [4], that have failed to distribute the gains of economic growth equitably. While research has shown that higher socio-economic status results in improvements in population health [5, 6], the effects have not necessarily occurred equitably and, some segments of the population often gain more than others. There is evidence that suggests that inequalities in various aspects of health have persisted in many developing countries and are often to the disadvantage of the poor and most vulnerable. For example, [7] highlight the persistence of inequalities in stunting in Nigeria and Bangladesh, despite a decrease in overall stunting rates. The poorest and those living in rural areas continued to be disproportionately affected, though the article also showed some evidence that the gap in other countries, such as Brazil, was narrowing [7]. Studies in South Africa have also shown that inequalities in stunting have reduced but have not been eradicated [8, 9].

In this paper, we use data from three African countries that graduated from low-income to lower middle-income status within the last decade (Ghana, Kenya and Zambia) to examine levels and trends in stunting across income groups and geographical locations. Stunting, low height for age, is an indicator of linear growth in children. It is regarded as an accurate measure of long-term malnutrition [9–11], because it is not as sensitive to temporary changes in food consumption as other measures of malnutrition like wasting and underweight [9]. Cumulative long-term investments in child health and nutrition, as well as other broader social policies for poverty reduction, are expected to have a positive impact on child growth and development and, subsequently, lead to a decline in child stunting. Thus, stunting is the most appropriate measure of malnutrition that can be used to assess the possible impacts of long-term economic changes on child outcomes and household wellbeing.

### The nature and effects of childhood malnutrition

Child malnutrition can take three forms: undernutrition, overnutrition and micronutrient deficiencies.^1^ Undernutrition occurs due to disease and insufficient food intake, and is manifested through stunting, wasting or underweight [12]. Overnutrition, on the other hand, occurs due lack of physical activity and excessive intake of unhealthy foods, and is manifested through overweight and obesity [13]. Insufficient intake of essential micro-nutrients results in micronutrient deficiencies such as iron and Vitamin A deficiencies [14].

All forms of malnutrition have detrimental effects on a child’s growth and development, especially in the early years life. The fastest growth of a child’s brain and physiology occurs in the first two years of life, making this period crucial for cognitive and motor development. Some studies have revealed that, in prioritising their energies, malnourished children have fewer calories to allocate to their physical, emotional and intellectual development, resulting in reduced absorption and learning [15, 16]. There are various other studies that have highlighted the negative impacts of malnutrition on a child’s cognitive development [7, 17–19]. Children who are malnourished are more likely to miss school classes [20–22], repeat grades or drop out of school [23, 24].

In addition, children who are malnourished are susceptible to diseases, and those who suffer from severe malnutrition are at risk of death [25]. However, the adverse effects of malnutrition are not limited to the early years of life, but continue into later years of childhood and persist in adulthood if not addressed. Research has also shown that children who are malnourished are at a higher risk of suffering from chronic and non-communicable diseases later in life and that labour productivity in adulthood is also affected [10, 26].

The Sustainable Development Goals (SDGs), which replaced the Millennium Development Goals (MDGs) after 2015, place a strong emphasis on eliminating hunger and reducing all forms of malnutrition. Over the years, there has been progress made in reducing extreme poverty and increasing access to education, but poor health and nutritional outcomes are widespread across Africa and other low-income regions. Globally, 52 million children under 5 years suffered from wasting in 2016, while stunting affected approximately 155 million children under 5 years [27]. Trend analysis shows that stunting levels in Africa declined marginally over a 15-year period: 31% of children under five were stunted in 2016, down from 38% in 2000 [27] . In comparison, stunting rates in Asia fell by 14% points over the same period [27].

In Ghana, Zambia and Kenya, the prevalence of stunting is higher than other forms of malnutrition, but evidence suggests that some progress is being made [28]. Stunting rates across the three countries have reduced over the years, an indication of progress and a reflection of a culmination of health system reforms and implementation of policies to reduce poverty, food insecurity and poor health. However, progress should not just be measured by reductions in stunting levels but should also consider the nature and distribution of stunting. As with all population-based indicators, variations in stunting are likely to exist across and within groups, with some populations being more negatively affected than others. Persistence of these variations, combined with the long-term nature of stunting, may slow down or hamper efforts to eliminate stunting.

### Economic Progress and inequalities in child stunting

The UNICEF conceptual framework for malnutrition classifies the causes of poor nutritional outcomes into three levels: immediate, underlying and basic [12, 29, 30]. Immediate causes, which occur at the individual level, include poor dietary intake and disease, and directly influence the nutritional status of a child [12, 29]. The underlying causes are directly linked to economic conditions at micro (household) and macro levels and generate, as well as, sustain the immediate causes of malnutrition [2, 12, 29].

At the micro level, poverty, household food insecurity, and social exclusion result in inadequate dietary intake, the inability of households to provide and sustain adequate care, and absence of healthy living environments. On the other hand, increases in household incomes and reduction in poverty levels have been linked to a higher caloric consumption [31, 32], while provision of adequate care and exposure to safe and healthy environments reduce the incidence, duration and severity of disease [33–35]. Efforts to reduce malnutrition should thus include policies and programmes that target increase in household income and improved access to food, basic living conditions and proper healthcare. An increase in economic growth can, therefore, result in a reduction in malnutrition rates if the increase leads to availability of opportunities that contribute to improvements in household welfare. Economic growth, which provides needed funds for national development, can contribute to improvements in the macro environment that is linked to malnutrition. Through increased investment in public health systems, timely preventive and curative healthcare can be made available to households [36, 37]. In addition, investments in education can have positive impacts on a child’s nutritional status, as better-educated caregivers are more likely to make better choices on child feeding and care [36]. Increased economic growth, and the ability to tax, also provides revenues that can be used to finance social protection programmes aimed at combating malnutrition, an example being cash grants targeted at children living in poor households.

In theory, Ghana, Zambia and Kenya, countries that graduated to MIC status over the last decade, should have recorded improvements in household welfare and nutritional status of children. Ghana’s graduation occurred in 2011 and has been attributed to a combination of rapid economic growth and a GDP rebasing [38]. Zambia’s graduation also took place in 2011, mainly due to improved macroeconomic environment including increases in foreign direct investments, economic growth and foreign aid driven interventions [39]. Kenya’s graduation was also driven by increased economic growth and a rebasing of the country’s GDP in 2014 [40]. National estimates from the three countries suggest that poverty and malnutrition rates have reduced. However, while increase in national incomes is a necessary condition, it is not sufficient for improved welfare. It is also important to consider the nature of the distribution of economic opportunities created by the expansion of an economy. While national incomes may increase, there are many who do not necessarily participate in the economic opportunities created, and so their socio-economic status do not improve. As a result, they and their children remain at risk of malnutrition. Thus, with increased economic growth, there are concerns around inequity and inequality that must be considered.

Inequity in this context refers to the exhibition of differences in the quality of health and access to health care across different population groups, which occur because of unequal economic and social conditions that are systemic and avoidable [41]. While disparities in health status between groups are inequalities [42], they are considered inequities if the differences are of preventable nature and are thus deemed unfair and unjust. In many instances, conditions that drive health inequities are neither natural nor inevitable but are consequences of public policies. Example of inequities include differences in presence of preventable diseases, access to and utilisation of healthcare, and health outcomes [43]. These differences are typically observed across geographical locations, race, ethnicity and socio-economic groups. In the case of malnutrition, higher levels are often seen in children living in rural areas relative to those in urban areas. Malnutrition rates are also higher amongst children living in households with poorer socio-economic status, compared to those in higher socio-economic groups. While these disparities may be considered inequalities, they are in fact inequities because they occur due to economic and social conditions that are avoidable.

Because child nutritional outcomes vary across groups, strategies to eliminate poor nutritional outcomes require differentiated approaches. There should be specific interventions that target those most affected by malnutrition to ensure that, as national incomes increase, inequalities in nutritional outcomes do not also persist or expand. To properly design such strategies, there is need for evidence on the nature of childhood malnutrition and the inequalities that exist across different groups. In this study, we focus on examining disparities that exist in stunting levels in Ghana, Kenya and Zambia, countries which appear to have made considerable progress in national growth and development.

While there are studies that have examined health inequities in the three countries, they are dated and have not made use of more recent national-level datasets. Some studies have also used health indicators, such as access to healthcare, to assess inequalities rather than nutrition indicators like stunting. In observing trends and nature of inequalities in stunting, we aim to provide evidence that can be used to inform design of strategies and interventions that are most suitable for combatting stunting levels in the three countries. We also aim to provide a deeper understanding of the effects of inequitable distribution of growth on children’s wellbeing. While our study focuses on three countries, it will provide insights into the nature of Africa’s persistent stunting problem and offer recommendations that are applicable to other countries with similar trajectories.

## Methods

### Study area

Ghana, Kenya and Zambia are agrarian and resource-dependent economies, and all graduated to middle-income country status within the last seven years. Despite the relatively high levels of GNI per capita of $3839, $3464 and $2881 for Ghana, Zambia and Kenya respectively (see Table 1), available data shows that poverty and social deprivation remain a challenge for all three economies. In fact, all three countries have maintained the characteristics of low-income economies and have high poverty levels.^2^ National statistics show that 24.2% of Ghana’s population lives below the national upper poverty line [44]. Poverty in Ghana has been characterised as regional and spatial; majority of the country’s poor live in rural areas where poverty rates are 37.9%, compared to urban poverty rates of 10.6% [44]. In Zambia, poverty affects 54.4% of the population, and is higher in rural areas (76.6%) compared to urban areas (23.4%) [45]. Most recent statistics from Kenya shows that 36.1% of the population lives below the country’s overall poverty line [46]. When rural and urban comparisons are made, poverty levels are found to be higher in the rural areas of the country: 40.1% of the population in rural areas lives in poverty, compared to 27.5% in the peri-urban areas and 29.4% in the core urban areas [46].

**Table 1:** Summary of selected poverty and health indicators

Country	Ghana	Zambia	Kenya
Human Development index	0.579	0.579	0.555
Life expectancy (years)	61.5	60.8	62.2
GNI per capita (PPP $)	3839	3464	2881
GINI	42.8 (2005)	55.6 (2010)	48.5 (2005)
Maternal mortality rate (per 100,000 live births)	319	224	510
Infant mortality rate (per 1000 live births)	42.8	43.3	35.5
Under-five mortality (per 1000 live births)	61.6	64	49.4
HIV/AIDS prevalence (% aged 15–49 years)	1.6	12.9	5.9

Unless otherwise specified, statistics are for 2015

Source: United Nations Development Programme - Human Development Report 2016

Table 1 shows statistics for select health and economic indicators for Ghana, Zambia and Kenya. Included is each country’s Human Development Index (HDI), a composite index with three dimensions of development: life expectancy, knowledge and decent standard of living [47]. All three countries are classified as having medium human development (0.555 to 0.579). For comparison purposes, the country with the highest index in the world is Norway with an HDI of 0.949, while Botswana’s index (0.698) is highest in sub-Saharan Africa. Amongst the three countries, under-five mortality rate is highest in Zambia followed by Ghana and Kenya. The infant mortality rate is also highest in Zambia, while Kenya’s maternal mortality rate is highest across the three countries. Zambia has the highest level of inequality, measured using a gini coefficient, but the data on economic inequality for the three countries, especially Ghana and Kenya, are dated so we are unable to provide recent estimates on the extent of inequalities in the two countries.

### Data source

The study makes use of data from Demographic and Health Surveys (DHS) conducted between 2001/02 and 2014: 2003, 2008 and 2014 in Ghana, 2001, 2007 and 2013/2014 in Zambia, and 2003, 2008/2009 and 2014 in Kenya. In all three countries, the DHSs were carried out by the respective national statistical agencies with technical support from ICF International through the DHS Program. The 2014 surveys in Ghana and Kenya were the sixth iteration of DHSs since inception in 1988 (Ghana) and 1989 (Kenya). Zambia’s 2013 survey was the fifth since the country first carried out a DHS in 1992. These nationally representative surveys are designed to provide data that can be used to monitor countries’ population, anthropometric, health and socio-economic indicators. DHSs adopts a two-stage stratified cluster sampling design, with Enumeration Areas (or clusters) selected during the first stage and households chosen at the second stage.

In Ghana, the 2014 DHS earmarked a sample of 12,831 households for interviews and achieved a response rate of 98.5% [48], while the 2008 survey realised a response rate of 98.9% out of a sample of 12,323 households, and the 2003 survey realised a response rate of 98.7% from 6333 sampled households [49, 50]. The three Zambian surveys both achieved a response rate of 98% from a sample of 7260 in 2001/02, 7326 households in 2007, and 16,258 households in 2013 [51–53]. The 2003 Kenyan survey had a response rate of 96.3% (from a sample of 8889 households), and 2008 survey had a response rate of 97.7% from 9936 households [54, 55]. In 2014, the number of households sampled increased significantly to 39,679, and the survey recorded a 99% response rate [56].

The DHS administers standardised questionnaires and variables across countries, making the survey data suitable for cross-country comparisons. While the DHS years do not necessarily match across the three countries we have included in this study, the variation is small and any differences resulting from variation in years is expected to be minimal, especially for cross-sectional survey data. Our study has been carried out with the assumption that the data from surveys in adjacent years are comparable.

### Analysing techniques and variables

We use the statistical software STATA (version 12) for analysis of the datasets. Anthropometric data (heights and weights) for children aged under 5 years were converted to Z-scores, based on 2006 WHO Child Growth Standards [57, 58]. We used height for age (HAZ), weight for age (WAZ) and height for weight (WHZ) Z scores, to generate three malnutrition indicators: stunting, underweight and wasting respectively. Stunting, our main variable of interest, is low height-for-age and reflects linear growth achieved at the age of measurement. Children are categorized as normal, moderately stunted (HAZ is between − 2 and − 3 standard deviations (SD) below the median) or severely stunted (HAZ is less than - 3 SD below the WHO child growth standards median) [11]. We classified all children whose height-for-age were less than − 2 SD as suffering from stunting. We also generated underweight and wasting estimates for purposes of providing a general overview of undernutrition. Underweight is defined as low weight for age and is caused by lack of food over the short term. Wasting, low height-for-weight, is an acute form of undernutrition and occurs due to insufficient food intake and infections. Children whose weight-for-age and weight-for-height is below − 2 SD of the WHO child growth standards median suffer from underweight and wasting respectively [11].

The Z scores were also used in the computation of inequality indices and generation of concentration curves. Concentration curves plot the cumulative percentage of a health variable against the cumulative percentage of the population, ranked by living standards, beginning with the poorest and ending with the richest. Concentration curves are useful when identifying whether socio-economic inequality in some health sector variable exists and varies across time and space [41, 59, 60]. Concentration indices enable the quantification of socio-economic related inequality in a health variable [61, 62]. This allows for assessment of the levels of inequality in the health variable (in this case, stunting) across socio-economic status which we measure using an asset index. The concentration index reflects twice the area between the concentration curve and the line of equality (the 45-degree line) and takes a value of zero for perfect equity. If the health variable of interest is undesirable, for example ill health or stunting, then a negative value of the concentration index means that ill health or stunting is higher among the poor.

The DHS does not collect data on income and expenditure, hence socio-economic status is measured using a wealth index [63] derived through the application of Principal Component Analysis (PCA) technique on a cumulative asset variable. This technique works on the principle that some unobserved variable, also called the latent variable, is correlated with a set of directly measured variables [64] which in this case are the asset variables. While income and expenditure parameters have traditionally been used to measure household economic status, the wealth indices used in our study are viable alternatives and are extremely important in the absence of income and expenditure data, as is the case with DHS. Since our analysis focuses on children aged under five years, we generated a sample of households containing at least one child aged under five years. In Ghana, the total (weighted) number of households with children under five years was 2746 in 2003, 2206 in 2008, and 2317 in 2014. In Kenya, the number was 3778 in 2003, 3782 in 2008, and 15,142 in 2014. In Zambia, the number of households with children under five years was 4275 in 2001, 4105 in 2007 and 9333 in 2013.

We computed a relative wealth index for the sub-sample (households with children under five years), based on households’ possession of durable goods, housing characteristics, and access to essential services. Similar to the DHS wealth index, we constructed PCAs for each dataset using the assets’ data, and weighted the score using each asset’s derived weight [65]. This results in standardised asset variables, whose scores are summed to give the final value (wealth index) [65, 66]. The correlations between our calculated wealth index, and that contained in the DHS files were very strong (> 0.99).

In addition to the wealth index, other variables included in this study are age and gender of the child, and geographical location.

## Results

### Descriptive analysis

In Table 2, we present descriptive statistics for select variables across the two surveys points for each of the countries. For numeric variables, the mean, minimum and maximum values are reported. For categorical variables, we report the percentages. In all the three countries, the most recent survey shows that the percentage of children under the 5 years living in rural areas is higher than those living in urban areas, though there has been an increase in the percentage of children living in urban areas over time. These differences are statistically significant (*P* < 0.001). Between 2001and 2014, the percentage of children living in urban areas in Kenya, Ghana and Zambia increased by 15.5, 12.6 and 2.6% respectively. Across gender, there were slight increases in the percentage of male children in all three countries over the three survey periods.

**Table 2:** Percentage of children under five years, by geographical location and gender

Country	Ghana			Zambia			Kenya		
Year	2003	2008	2014	2001	2007	2013	2003	2008	2014
n	3873	3128	3255	6581	6450	14,067	6332	6301	21,262
Rural	66.5%	61.1%	53.9%	68.7%	71.6%	66.1%	82.4%	83.1%	66.9%
Urban	33.5%	38.9%	46.1%	31.3%	28.4%	33.9%	17.6%	16.9%	33.1%
Male	50.3%	51.0%	52.3%	49.7%	49.5%	50.5%	50.0%	51.1%	50.7%
Female	49.7%	49.0%	47.6%	50.3%	50.5%	49.5%	50.0%	48.9%	49.3%

Source: DHS (2001, 2003, 2007,2008, 2013 and 2014). Authors’ calculations

**Fig. 1:**
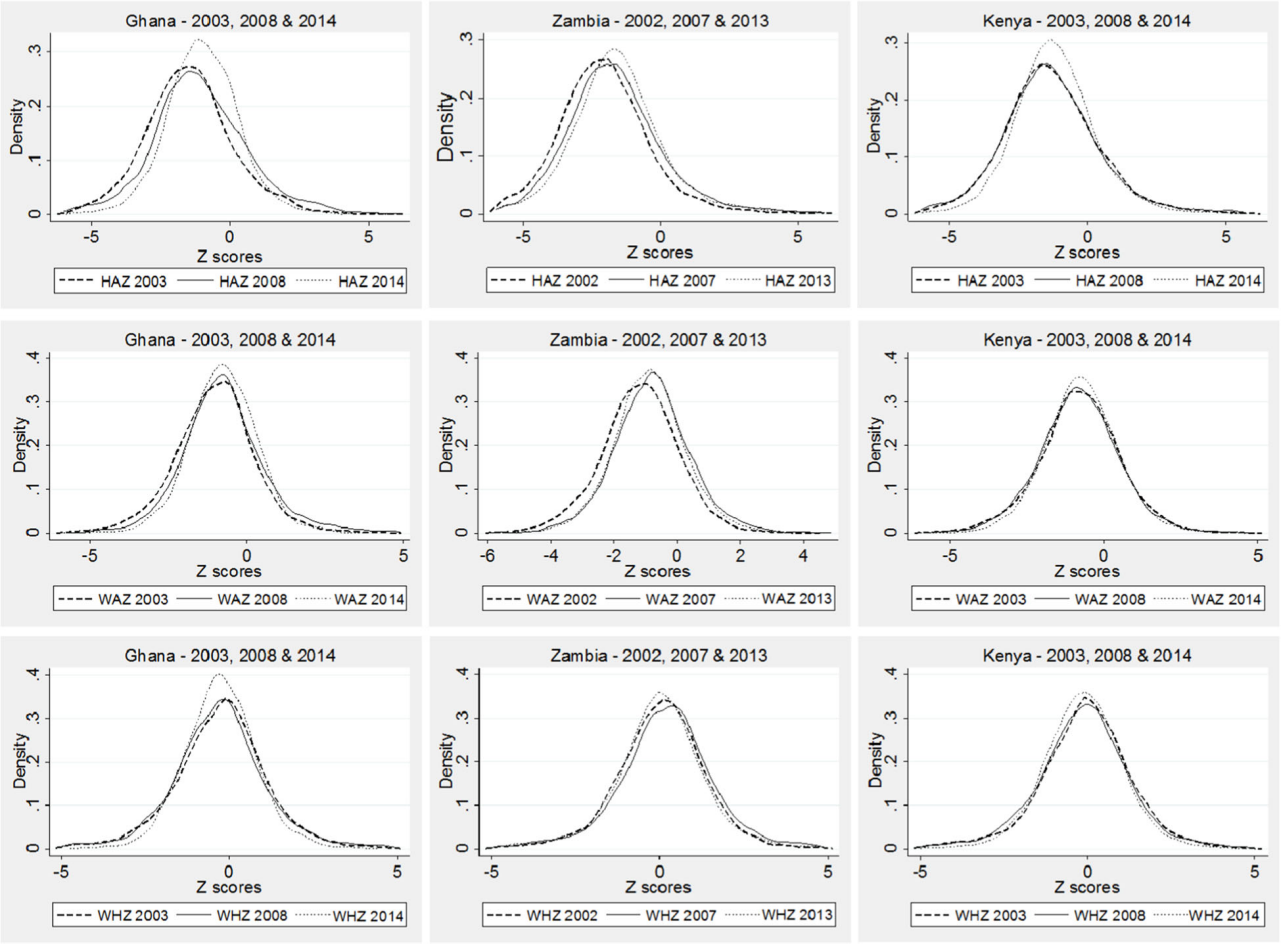
Kernel density estimations for Ghana, Zambia and Kenya, using HAZ, WAZ and WHZ. Source: DHS (2007,2008, 2013 and 2014). Authors’ calculations

The general trend observed across the three countries suggests increases in mean Z scores’ values in the three survey periods, except for the WAZ and WHZ in Zambia, and WHZ in Kenya. This is illustrated in the kernel density estimations shown in Fig. 1, which provide a visualization of the underlying distribution of the Z scores. Across all three countries, the biggest improvements in child anthropometric status was in HAZ. In all three graphs, the third surveys (2013/14) lines (represented by the dotted lines) are to the right of the initial surveys lines, showing that height-for-age Z scores improved over time. In absolute numbers, the mean HAZ increased from − 1.44 in 2003 to − 1.08 in 2008 and − 0.93 in 2014 for Ghana (*P* < 0.05), and from − 2.04 in 2001 to − 1.65 in 2007 and − 1.53 in 2013 for Zambia (*P* < 0.001). In the case of Kenya, there are no significant difference in HAZ between 2003 and 2008, but HAZ increased from − 1.31 in 2008 to − 1.16 in 2014 (*P* < 0.001).

In comparison, the mean WAZ increased from-1.02 in 2003 to 0.74 in 2008 for Ghana (P < 0.001) but no significant changes were observed between 2008 and 2014. In Zambia, mean WAZ increased from − 1.19 in 2001 to − 0.79 in 2007 but dropped to − 0.88 in 2013 for Zambia (*P* < 0.001). In Kenya, the mean WAZ increased from − 0.82 2008 to − 0.75 in 2014 (P < 0.001). There was no significant change in mean WHZ in Ghana across the three periods. WHZ remained unchanged in Kenya between 2008 and 2014, but dropped significantly in Zambia from 0.19 in 2007 to 0.08 in 2013 (*P* < 0.001).

### Levels of malnutrition in Ghana and Zambia

**Fig. 2:**
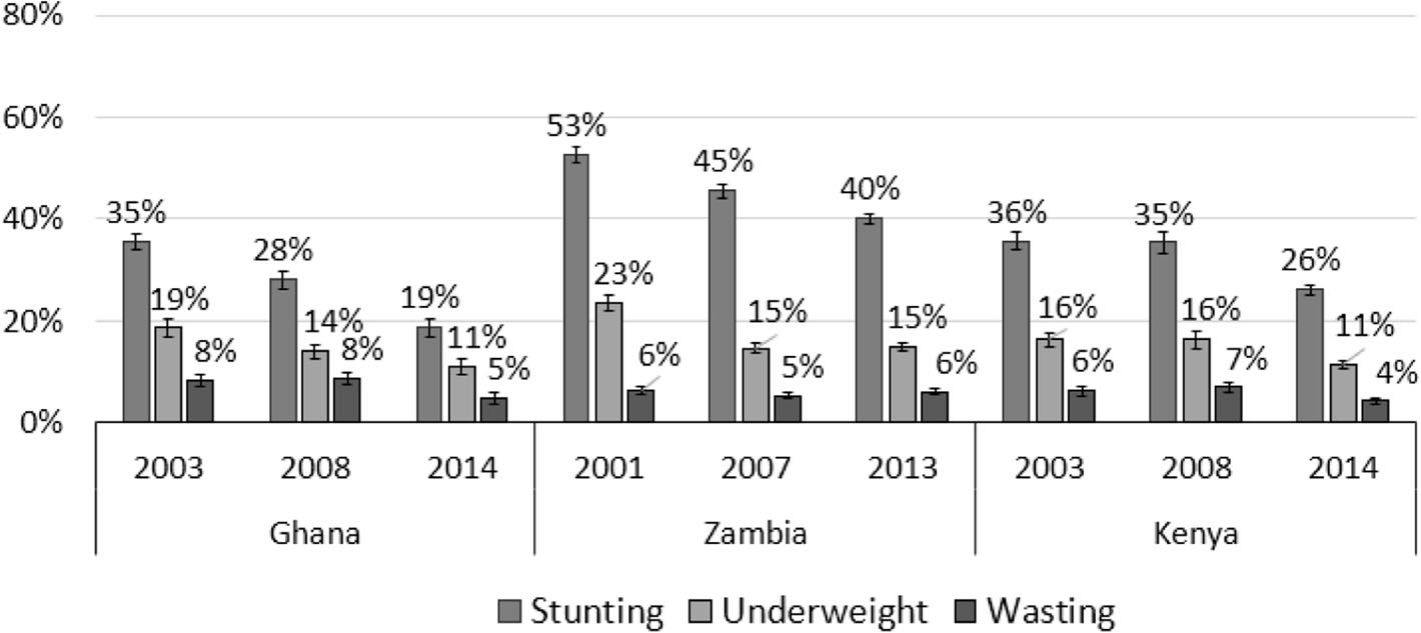
Levels of malnutrition in Ghana, Kenya and Zambia. Source: DHS (2007,2008, 2013 and 2014). Authors’ calculations

Figure 2 shows that malnutrition levels in Ghana declined significantly between 2003 and 2014. The reduction was greatest for stunting, which declined from 35 to 19%, while underweight and wasting dropped from 19 to 11%, and 8 to 5% respectively (*P* < 0.001). Unlike Ghana, trend analysis shows mixed results for Zambia. While stunting reduced from 53% in 2007 to 40% in 2013 (P < 0.001), and underweight rates from 23 to 15% (though remained unchanged between 2008 and 2014), there were hardly any changes in the prevalence of wasting over the period. In Kenya, the percentage of children who were stunted, underweight or wasted declined between 2003 and 2014. In 2003, 36% of children under five were stunted, 16% of children were underweight and 6% were wasted. This fell to 26% for stunting, 11% for underweight and 4% for wasting in 2014. These differences are statistically significant (*P* < 0.001). However, it is worth noting that there were minor changes in malnutrition rates in Kenya between 2003 and 2008.

Table 3 shows results from bivariate analyses of associations between stunting, and gender, location and wealth groups. We found that stunting was more prevalent in male children than in females in all three countries across the three periods. While the 2013/2014 levels indicate a decline in stunting over the years, male children remain most affected, compared to females. The differences across gender were statistically significant for Kenya and Zambia across the three periods (*P* < 0.001). Analyses show that stunting is more prevalent in rural areas than in urban areas in all three countries across all survey years (P < 0.001). While the results show a reduction over time in malnutrition levels for both rural and urban areas, we find a higher decrease in rural areas, compared to urban areas. In Ghana, the percentage of stunted children in rural areas declined by 18.5% points between 2003 and 2014, compared to 9.8%-point reduction in urban areas. In Zambia, rural stunting rates declined by 14.9% points between 2001 and 2014, compared to 6.8%-point decline in the urban areas. The differences in percentage-point reduction were smaller Kenya where there were no significant changes in stunting rates between 2003 and 2008, but between 2008 and 2014 they declined by 8.1% points in rural areas and 6.0% points in urban areas, showing a much smaller difference in reduction rates between geographical areas, compared to Ghana and Zambia.

**Table 3:** Stunting levels, by gender, geographical location and wealth quintiles

	Country	Ghana			Zambia			Kenya		
	Year	2003	2008	2014	2001	2007	2013	2003	2008	2014
Gender	Male	38.8%	29.7%	19.9%	54.6%	48.7%	42.4%	39.4%	37.3%	29.7%
	Female	32.2%	26.4%	17.9%	50.6%	42.3%	37.8%	31.8%	33.2%	22.3%
Area	Rural	41.1%	32.2%	22.6%	57.0%	48.2%	42.1%	36.8%	37.2%	29.1%
	Urban	24.4%	21.8%	14.6%	42.8%	39.8%	36.0%	29.7%	25.7%	19.7%
Wealth quintile	1, poorest	48.8%	36.5%	26.5%	58.4%	47.1%	46.7%	42.4%	43.7%	36.5%
	2, poorer	37.9%	31.2%	26.1%	60.3%	51.3%	43.7%	39.5%	42.1%	30.2%
	3, middle	38.9%	30.3%	16.2%	56.5%	48.1%	38.7%	36.6%	33.9%	26.6%
	4, richer	29.9%	22.2%	17.9%	48.6%	46.2%	39.8%	31.5%	29.5%	19.7%
	5, richest	18.2%	18.1%	5.5%	37.0%	35.3%	30.0%	25.5%	23.5%	13.1%

Source: DHS (2001,2003,2007,2008, 2013 and 2014). Authors’ calculations

A similar pattern is seen when we compare household wealth status and stunting in Ghana. In Ghana, the highest stunting rates were in the poorest households across the all three periods (*P* < 0.001). The percentage of children suffering from stunting in the poorest quintile was 48.8% in 2003, compared to 18.2% in the richest quintile. In 2014, all wealth quintiles in Ghana recorded lower stunting levels compared to 2003 and 2008. However, inequalities in nutritional status persisted over time, with the poorest households recording the highest levels of malnutrition. This is also the case with Zambia and Kenya, where children from poorest wealth quintiles have the highest rates of stunting for the three periods under consideration (P < 0.001). In the two countries, stunting rates declined significantly across all the quintiles between 2001, 2007 and 2013 (Zambia) and 2003, 2008 and 2014 (Kenya) though further analysis shows that inequalities in stunting in the two countries have persisted across the two periods. In fact, a comparison of Kenyan data (2003 vs 2014) shows that stunting rates amongst children living in the richer households (quintiles 4 and 5) declined by 12% points over that period, twice the rate of decline recorded amongst children living in households in the poorest quintile.

Stunting rates in Zambia are significantly higher compared to Ghana and Kenya, especially in the poorest wealth quintiles where, in 2013, close to half of the children in the poorest quintile (47%) were stunted, compared 30% of the children in the richest quintile which is higher than the overall stunting rates for Kenya and Ghana (26 and 19% respectively in 2014).

### Inequalities in stunting

**Fig. 3:**
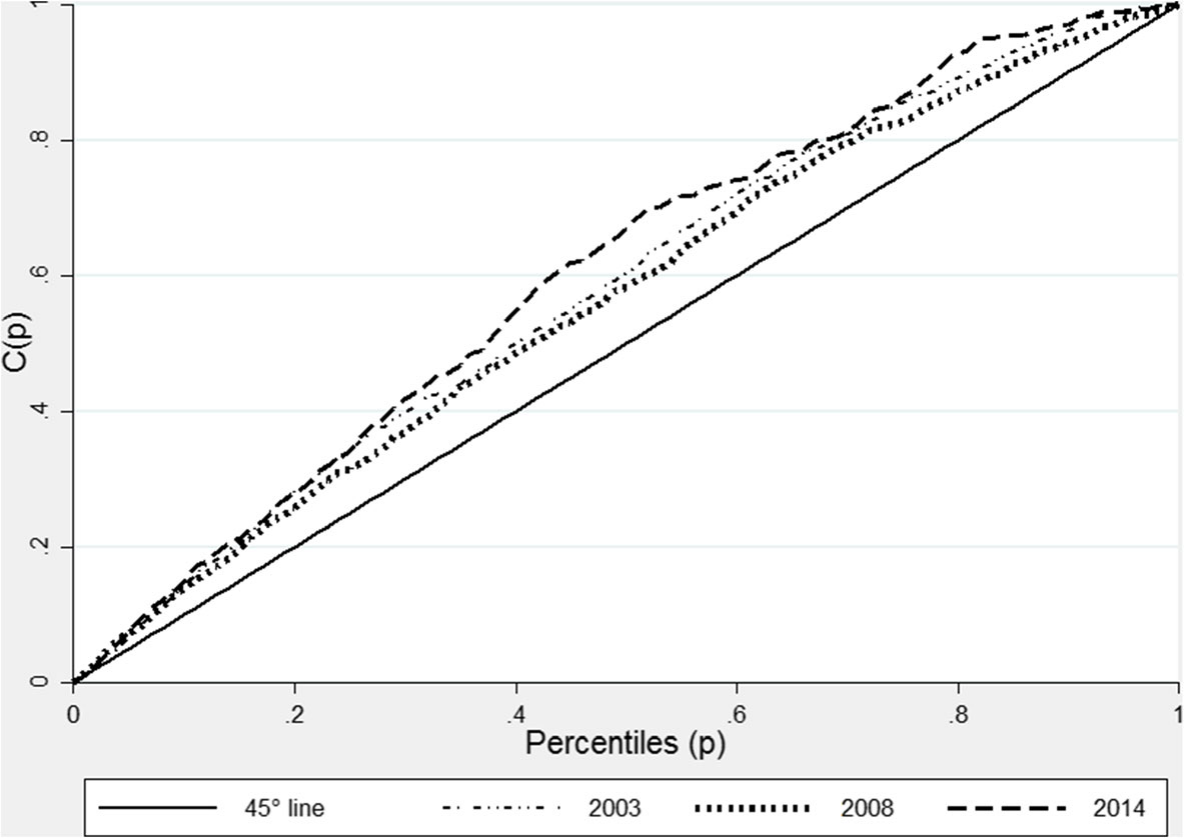
Stunting Concentrations curve for Ghana 2008 and 2014. Source: Ghana DHS 2008 and 2014. Authors’ calculations

In Fig. 3, we present curves showing stunting inequalities in Ghana in 2003, 2008 and 2014. We find that for the three surveys, stunting concentration curves lie above the 45-degree line of equality indicating that stunting levels have continuously and disproportionately affected the poor in the country. The outward shift in the concentration curve in 2014 suggests that inequalities in stunting have increased over time, despite a reduction in stunting levels between 2003 and 2014. This is confirmed in the corresponding concentration indices of − 0.15 in 2003 and − 0.18 in 2014 (Table 4). The negative signs in the indices show that inequalities have been more detrimental for the poor, compared to those who are better off.

**Table 4:** National Concentration index (stunting) - Ghana, Zambia and Kenya

Country	Year	Estimate	Standard error	[95% confidence interval]	
Ghana	2003	− 0.15	0.00	− 0.15	-0.15
	2008	−0.12	0.00	−0.12	− 0.12
	2014	−0.18	0.00	−0.18	−0.18
Zambia	2001	−0.07	0.01	−0.09	−0.05
	2007	−0.05	0.01	−0.07	−0.03
	2013	−0.08	0.01	−0.09	−0.06
Kenya	2003	−0.10	0.01	−0.12	−0.07
	2008	−0.11	0.01	−0.13	−0.08
	2014	−0.15	0.01	−0.17	−0.14

Source: DHS (2001, 2003, 2007,2008, 2013 and 2014). Authors’ calculations

**Fig. 4:**
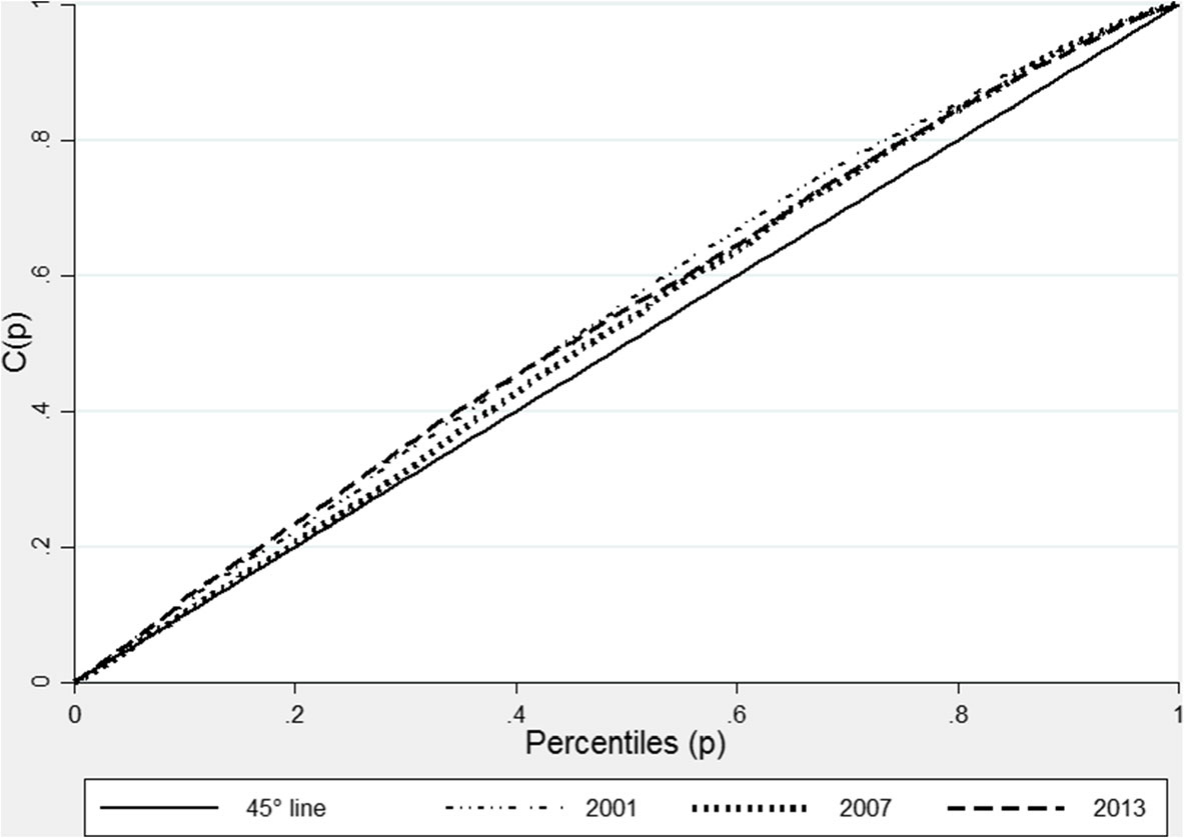
Stunting Concentration curve for Zambia 2007 and 2013. Source: Zambian DHS 2007 and 2013. Authors’ calculations

Concentration indices for Zambia, also reveal that in addition to the high levels of stunting in the country, inequalities in stunting levels have increased, especially when we compare 2007 and 2013 (see Table 4). The Zambian concentration curves shown in Fig. 4 are also much closer to the line of equality, compared to those of Ghana. However, as with Ghana, the concentration curves for Zambia all lie above the 45-degree line of equality illustrating that inequalities in stunting have disproportionately affected the poor. However, as shown in Fig. 4, the curves for the three surveys intercept showing that the increase in inequality was not clear in all socioeconomic categories.

**Fig. 5:**
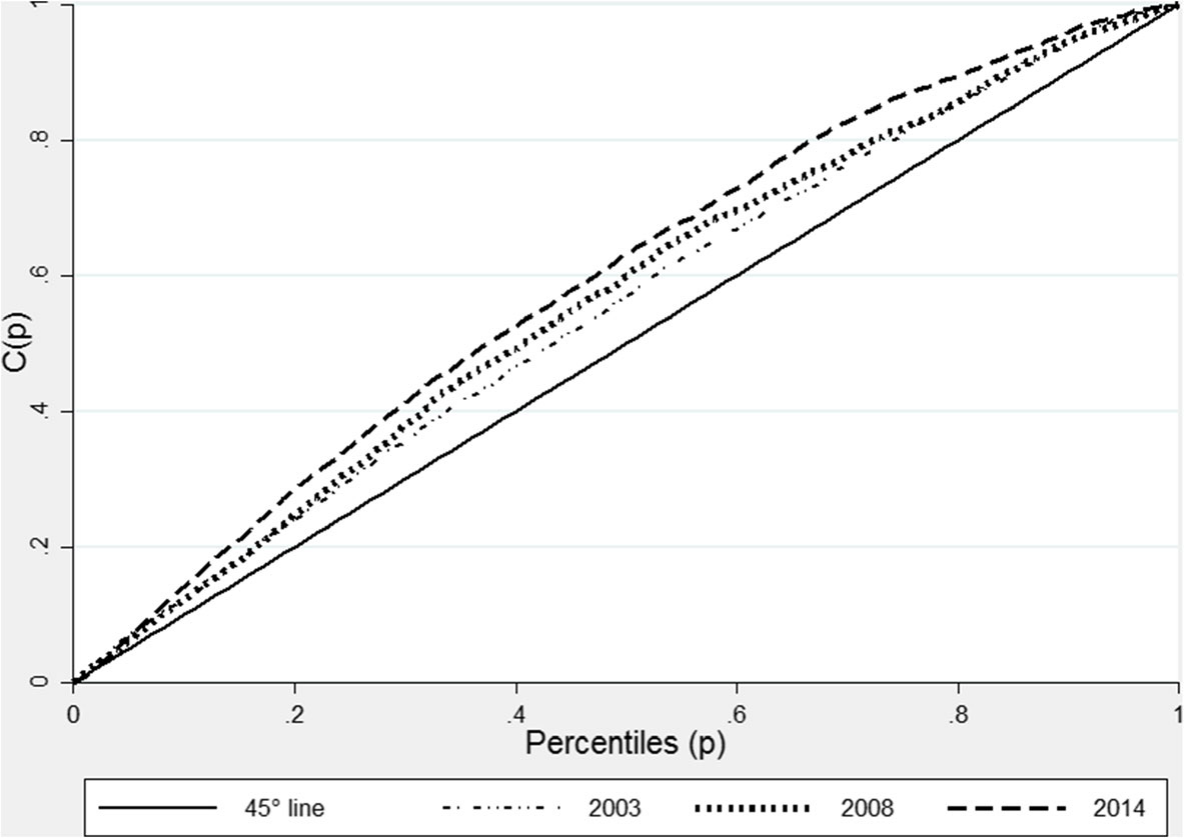
Stunting concentration curve for Kenya, 2008 and 2014. Source: Kenyan DHS 2008 and 2014. Authors’ calculations

For Kenya, the 2003, 2008 and 2014 concentration curves presented in Fig. 5 lie above the 45-degree line, indicating children from poor households are disproportionality affected by stunting. Despite a remarkable reduction in the levels of stunting between 2008 (35%) and 2014 (26%), inequalities in stunting appear to have worsened in 2014. These results are reinforced by concentration indices that are shown in Table 4: in 2014, the concentration index was −− 0.15, compared to − 0.11 in 2008.

### Rural/urban inequalities in stunting

To gain a better understanding of the patterns of stunting inequalities, we examined these inequalities in rural and urban areas for the two survey points,^3^ for all the three countries. The concentration indices for rural and urban areas for the three countries shows increase in inequalities, especially when we consider the first and third survey periods, and the second and third surveys. Inequalities appear to be greater in the urban areas, compared to the rural areas. The Ghanaian data show that between 2008 and 2014, the values in urban areas increased from − 0.16 to − 0.24 respectively, and − 0.04 to − 0.10 in rural areas over the same period (see Table 5). In Kenya, the inequalities in the urban areas appear to have worsened between the two periods: the concentration index in 2008 was − 0.14, compared to − 0.25 in 2014 (see Table 5).

**Table 5:** Rural and urban concentration indices for Ghana, Zambia and Kenya

Country	Year	Geographical area	Estimate	Standard Error	[95% confidence interval]	
	2003	Urban	−0.19	0.00	−0.19	−0.19
		Rural	−0.07	0.00	−0.07	−0.07
Ghana	2008	Urban	−0.16	0.05	−0.26	−0.07
		Rural	−0.04	0.02	−0.09	0.01
	2014	Urban	−0.24	0.04	−0.31	−0.16
		Rural	−0.10	0.02	−0.14	−0.06
	2001	Urban	−0.09	0.02	−0.13	−0.05
		Rural	−0.03	0.01	−0.04	−0.01
Zambia	2007	Urban	−0.11	0.03	−0.17	−0.05
		Rural	−0.01	0.01	−0.03	0.01
	2013	Urban	−0.11	0.02	−0.14	−0.07
		Rural	−0.04	0.01	−0.06	−0.03
	2003	Urban	−0.19	0.04	−0.26	−0.12
		Rural	−0.07	0.01	−0.10	−0.05
Kenya	2008	Urban	−0.14	0.05	−0.24	−0.04
		Rural	−0.07	0.01	−0.10	−0.04
	2014	Urban	−0.25	0.03	−0.30	−0.20
		Rural	−0.08	0.01	−0.10	−0.07

Source: DHS (2001,2003,2007,2008, 2013 and 2014). Authors’ calculations

**Fig. 6:**
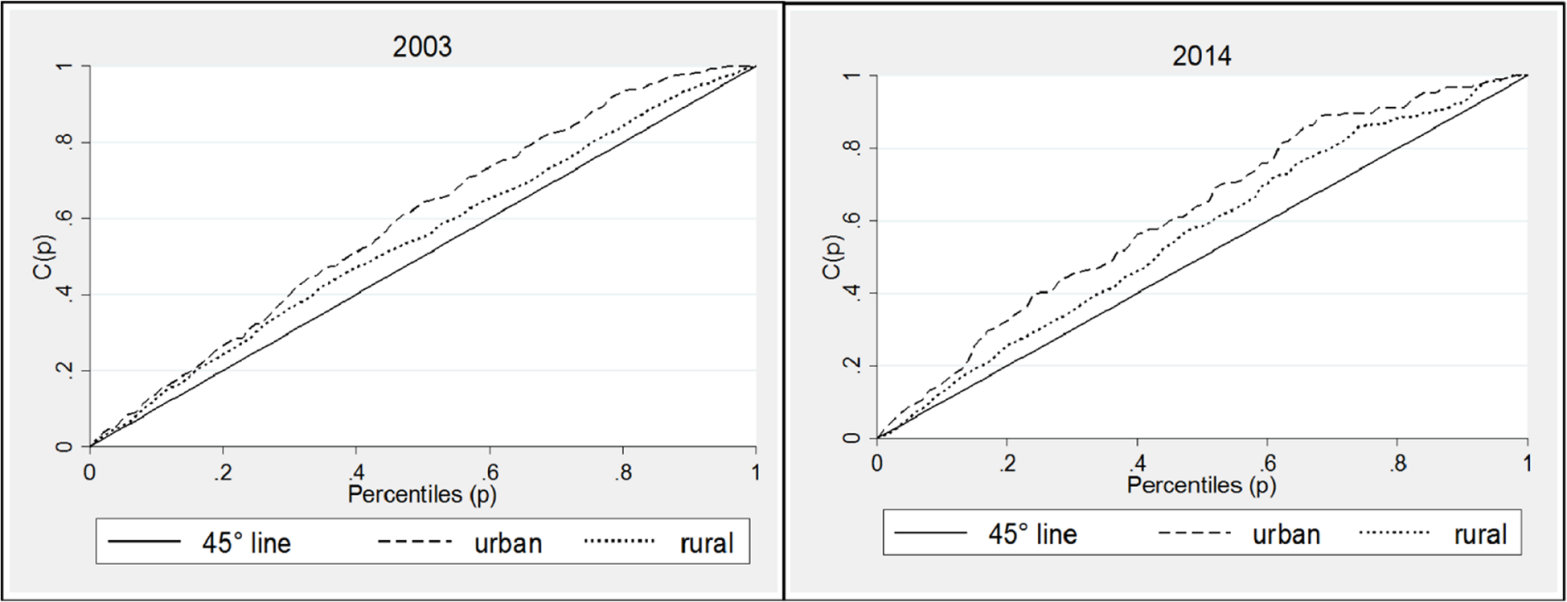
Urban and rural concentration curves for stunting in Ghana. Source: DHS (2008 and 2014). Authors’ calculations

**Fig. 7:**
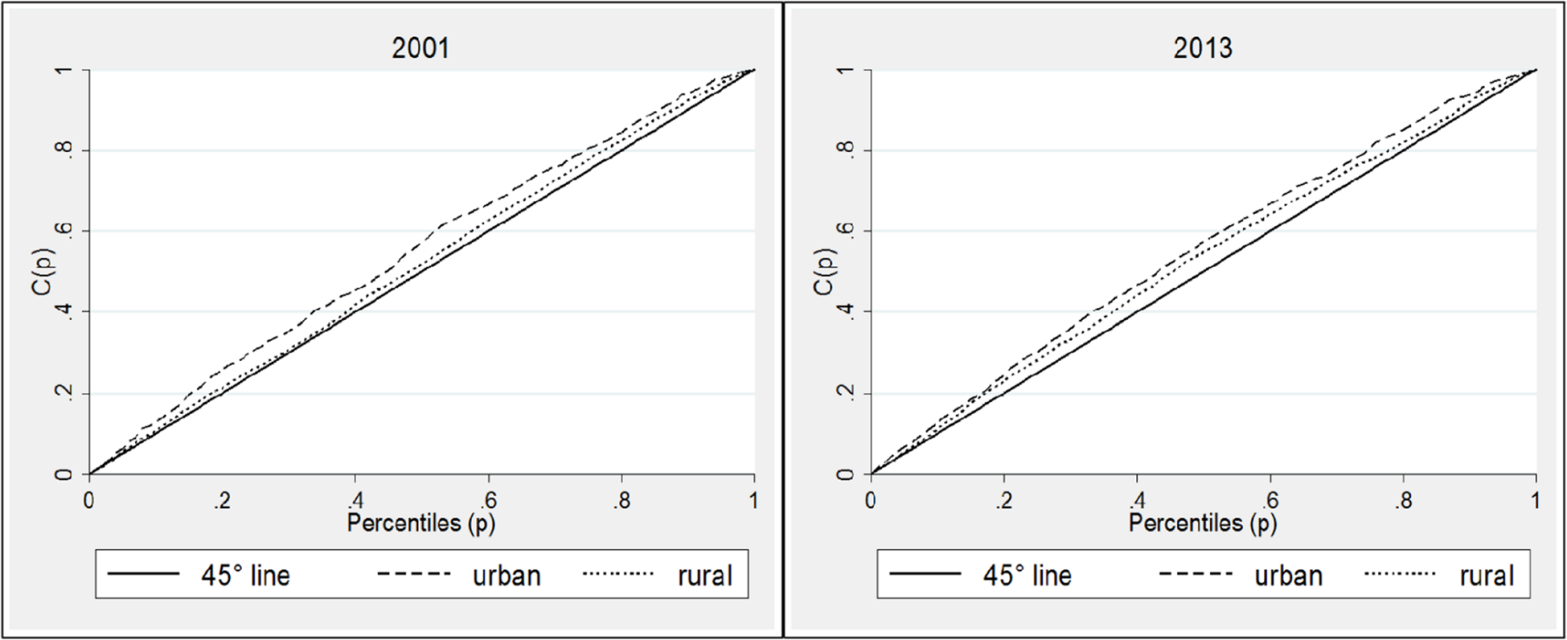
Urban and rural concentration curves for stunting in Zambia. Source: DHS (2007 and 2013) DHS. Authors’ calculations

**Fig. 8:**
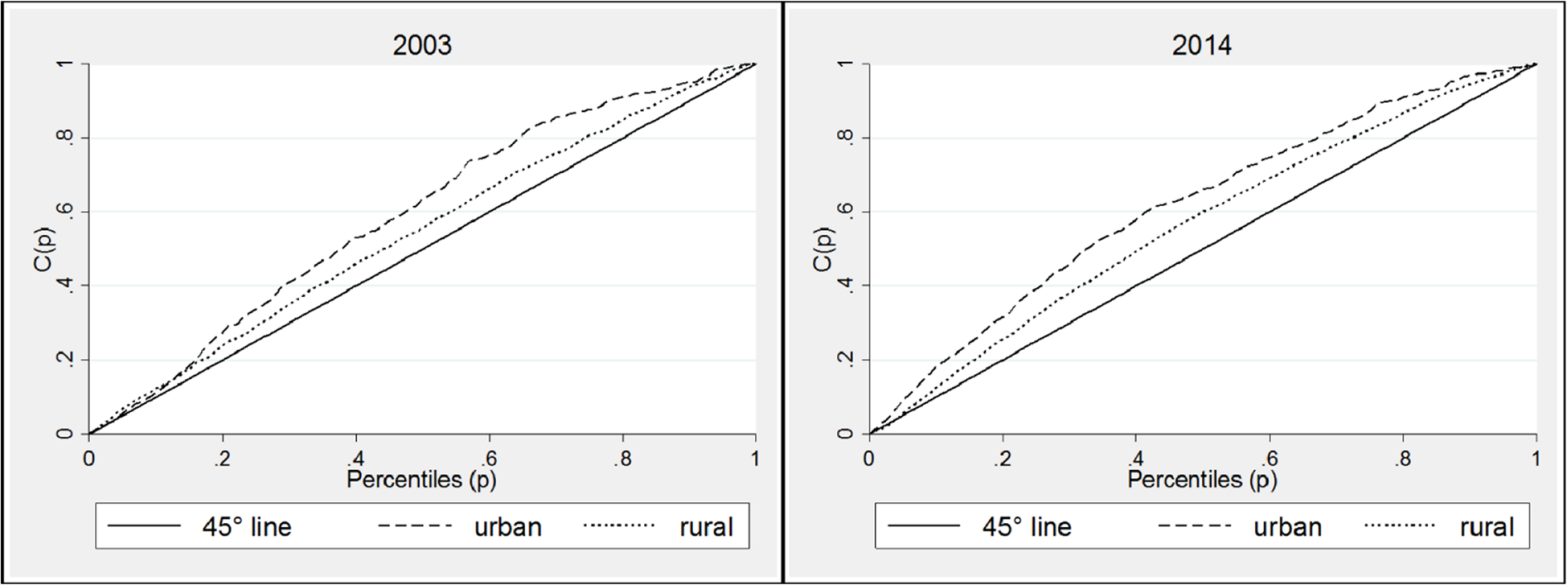
Urban and rural concentration curves for stunting in Kenya. Source: DHS (2008 and 2014). Authors’ calculations

Concentration curves for the three countries confirm the patterns seen in the analysis of the concentration indices. In Ghana, for both urban and rural areas, the concentration curves lie above the equality lines for both 2003 and 2014 curves, showing that inequalities have disproportionately affected children living in the poorest households (Fig. 6). Analysis of Zambian data shows similar patterns; the concentration curve for rural and urban areas (Fig. 7) show that inequalities in stunting were higher in urban areas than rural areas in 2001 and 2013, though in the later inequalities appear to have increased in rural areas. For both years, however, stunting has been concentrated amongst the poor households, as indicated by the concentration curves lying wholly above the line of equality, and the corresponding indices which are presented in Table 5. Kenyan concentration curves (Fig. 8) shows that stunting inequalities are greater in the urban areas, compared to the rural areas for both 2003 and 2014.

## Discussion

The nutritional status of children under-five is an established measure of child health and general household well-being. In this study, we examined the prevalence of stunting (the most prominent form of malnutrition) and inequalities in stunting in Ghana, Kenya and Zambia, three African countries that graduated from low income status to middle income status during the last decade. Our aim was to assess trends in stunting levels, given increases in national income levels. Our findings indicate that there were decreases in stunting rates in all the three countries over the periods examined. The decreases were higher in Ghana and Zambia, which saw stunting rates reduce by 16.8%-points and 12.5%-points respectively, while in Kenya it reduced by 9.6% points. For Ghana and Kenya, the reduction was more apparent between 2008 and 2014, compared to Zambia where the decline was higher between 2001 and 2007, compared to 2007 /13 period. Sex-specific analyses reveal significant declines in stunting for males and females, but stunting rates have remained consistently higher among male children, compared to females. We also found that despite economic progress and reductions in stunting over the time, all three countries have consistently recorded higher levels of stunting in rural areas, compared to urban areas. Recent studies have shown that despite increases in poverty levels in urban areas, rural areas still bear a higher share of poverty and have poorer access to basic services [45, 46, 67].

**Fig. 9:**
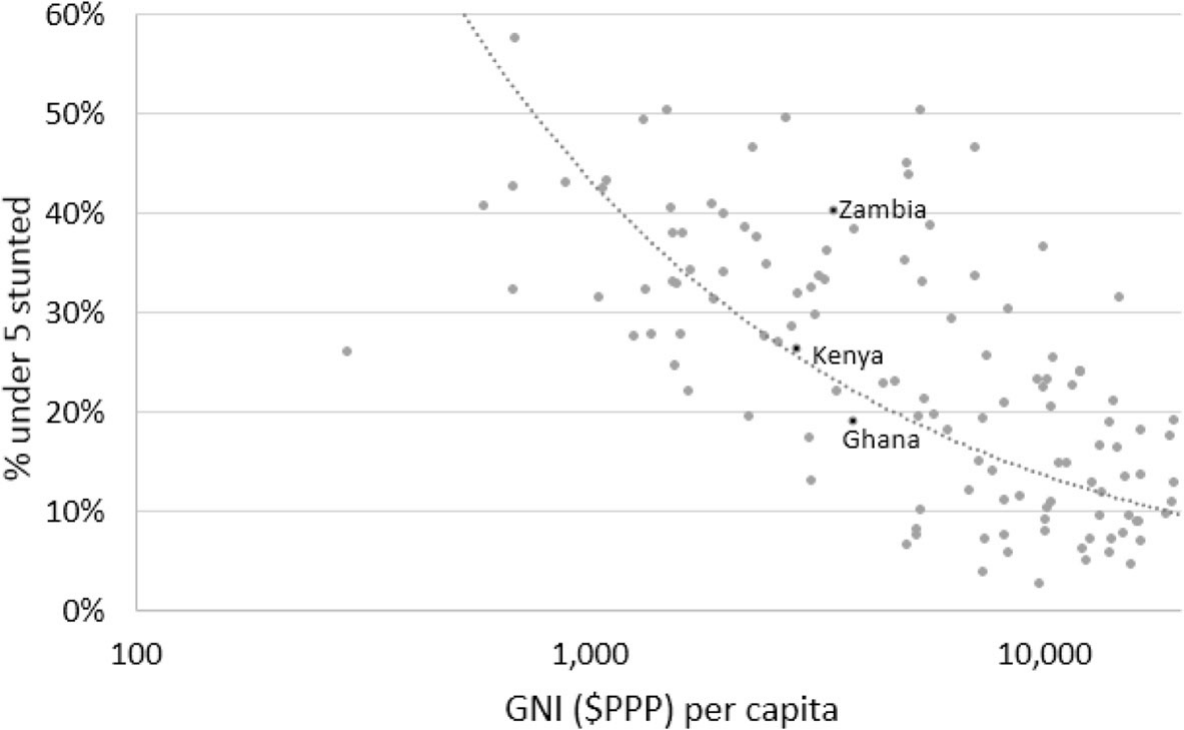
Stunting rates, by GNI ($PPP) per capita. Source: United Nations Development Programme - Human Development Report 2016

As shown in Fig. 9, which compares the GNI per capita ($ PPP 2011) and stunting rates for children under five for over 154 countries with available data,^4^ countries with the highest GNI per capita also have the lowest stunting rates. Ghana, which has the highest GNI per capita of the three countries included in this study, has the lowest stunting rate. However, as the graph shows, some countries with relatively high GNI per capita also have high stunting rates, an example being Zambia where 40% of children under five years are stunted. Another example is South Africa, with a reported GNI per capita of $12,087 [47], and a stunting rate of 27% for children under five [68].

National statistics often mask inequalities that exist across and between groups, so our study focused on examining disparities across geographical locations and between groups of different socio-economic status. In line with other studies [8, 69], we found that the poorest households have the highest stunting rates. Our analyses of the most recent datasets show that in Ghana, stunting rates in the wealthiest quintile was three times lower than that of the lowest quintile and in Kenya, stunting rates amongst children living in the poorest households were 2.7 times that of those living in the wealthiest quintile. These differences appear to have worsened since the 2007/2008 surveys were conducted. In Zambia, we found that stunting rates were very high across all wealth groups, and the prevalence in the poorest wealth quintile was 1.6 times that of the richest wealth quintile in 2013. The inequalities amongst the richest and poorest wealth quintiles increased marginally between 2007 and 2013. Further analyses using concentration curves and indices show that inequalities in stunting persisted and worsened over time in all three countries, despite reduction in stunting rates. In Ghana and Kenya, inequalities were higher in the urban areas, and increased between 2008 and 2014. While the inequalities in Zambia were high across urban and rural areas, they increased in rural areas between 2007 and 2013 but remained unchanged in the urban areas.

In all the three countries, several programmes have been initiated to improve child nutrition and health outcomes and these appear to have culminated in a reduction in stunting, suggesting some improvement in long-term nutritional status of children. These programmes include mandatory staple fortification, vitamin A supplementation, national school feeding schemes, and nutrition based outreach programmes [70–72]. They were implemented to reduce the prevalence of malnutrition and improve access to health care for malnourished children across the geographic and socioeconomic divide. The significant decline in stunting levels in Kenya and Ghana suggest that these programmes are having a positive impact on child nutritional outcomes. The levels of underweight and wasting in Ghana and Kenya also fell between 2008 and 2014. The two countries have also recorded improvements in other socio-economic indicators of wellbeing. Headcount poverty in Ghana decreased from 56.5% in 1992 to 24.2% in 2013 [44, 67]. In Kenya, recently released findings from a household budget survey shows that overall poverty levels decreased by 10% points over a 10-year period: 2005/2006 in 46.8 to 36.1% in 2015/2016 [46].

Zambia’s stunting rates are very high (40% in 2013) despite a 12.5%-points between 2001 and 2013. Stunting rates remain significantly high across all wealth groups, despite the country having a higher GNI per capita than Kenya where stunting rates are at 26% in 2014. Poverty in Zambia also remains high; extreme poverty levels reduced from 58% in 1991 to 42.3% in 2010 [73] and was reportedly 40.8% in 2015 [45]. The country’s gini coefficient is one of the highest in the world [74]. However, Zambia is one of the few countries where the annual rate of reduction for stunting is above 2% [28]. The country’s budgetary allocation to nutrition-specific and nutrition-sensitive interventions is the lowest of the three countries included in our study: 2017 analysis showed that the country’s budgetary share to these interventions was less than 1%, compared to 4.3% in Kenya and 4.8% in Ghana [75]. However, the country appears to have increased spending on nutrition sensitive interventions between 2013 and 2015 by 50% [75].

All three countries have implemented cash transfer programmes for poor households and orphan and vulnerable children, but these cover a small proportion of poor children, especially when compared to countries like South Africa where over two thirds of all children are recipients of the Child Support Grant [76]. This raises the risk of exclusion for children most in need of income support, including those who are malnourished. To make significant progress to achieve the SDG target of eliminating stunting by 2030, all three countries need to increase investments in food security and nutrition and implement programmes to reduce poverty and improve children’s living conditions.

## Conclusions and recommendation

The cumulative effects of economic growth, poverty reduction and other polices to improve health and nutrition appear to have been translated into reductions in stunting rates in Kenya, Ghana and Zambia. However, inequalities have persisted and appear to be widening over time. The poor and those living in rural areas are most affected.

It is vital that all citizens benefit from improved economic conditions, especially after the attainment of MIC status. This can be done through enhancements in quality healthcare and adequate dietary intake, and improvements in household living conditions and socio-economic status. Policies for poverty reduction, and income support for the poor, will enhance the purchasing power of poor urban and rural households. Long-term policies should also target increases in labour market participation and, in the interim, cash transfers to poor households can be used to provide crucial supplementary income that can ensure economic access food and other necessities. Additionally, there is need for implementation of food policies that focus on making nutritious foods available and affordable for poor households. These could include making fortified staples more available, and eliminating value added taxes for nutritious foods. Attaining universal health coverage is also essential in ensuring that all people, especially those who are poor, have access to proper healthcare to enable them lead healthy and productive lives. If done right, implementation of these policies will result in sustained improvements in child nutritional outcomes.

## Abbreviations

DHS: Demographic and health survey
GDP: Gross Domestic Product
GNI: Gross national income
HAZ: Height for age
HDI: Human development index
MIC: Middle income country
PCA: Principal component analysis
PPP: Purchasing power parity
SD: Standard deviation
SDGs: Sustainable development goals
UNDP: United Nations Development Programme
UNICEF: United nations children’s fund
WAZ: Weight for age
WHO: World Health Organization
WHZ: Weight for Height

## Footnotes

1 http://www.who.int/news-room/fact-sheets/detail/malnutrition.

2 In terms of population living below the international poverty line ($1.90 a day), the variations in data years across the three countries makes comparisons difficult. However, the most recent data shows that in 2012, 12% of the population in Ghana lived below $1.90 a day (2011 PPP). In Zambia, 57.5% of the population lived below $1.90 a day in 2015, while in Kenya the most recent available data (2015), shows that 36.8% of the population lives below $1.90 a day (2011 PPP). Statistics are available here: http://povertydata.worldbank.org/poverty/home.

3 The DHS geographical classifications are based on individual countries’ statistical agencies’ definition of rural and urban areas.

4 GNI $PPP data is for 2015. Stunting rates are based on the most recent available datasets, ranging from 2010 to 2015.
